# Chondrocytes differentiated from human induced pluripotent stem cells: Response to ionizing radiation

**DOI:** 10.1371/journal.pone.0205691

**Published:** 2018-10-23

**Authors:** Ewelina Stelcer, Katarzyna Kulcenty, Wiktoria Maria Suchorska

**Affiliations:** 1 Radiobiology Laboratory, Greater Poland Cancer Centre, Poznan, Poland; 2 The Postgraduate School of Molecular Medicine, Medical University of Warsaw, Warsaw, Poland; 3 Department of Electroradiology, Poznan University of Medical Sciences, Poznan, Poland; Northwestern University Feinberg School of Medicine, UNITED STATES

## Abstract

**Purpose:**

Data on the response of chondrocytes differentiated from hiPSCs (hiPSC-DCHs) to ionizing radiation (IR) are lacking. The aim of present study was to assess DNA damage response (DDR) mechanisms of IR-treated hiPSC-DCHs.

**Methods and materials:**

The following IR-response characteristics in irradiated hiPSC-DCHs were assessed: 1) the kinetics of DNA DSB formation; 2) activation of major DNA repair mechanisms; 3) cell cycle changes and 4) reactive oxygen species (ROS), level of key markers of apoptosis and senescence.

**Results:**

DNA DSBs were observed in 30% of the hiPSC-DCHs overall, and in 60% after high-dose (> 2 Gy) IR. Nevertheless, these cells displayed efficient DNA repair mechanisms, which reduced the DSBs over time until it reached 30% by activating key genes involved in homologous recombination and non-homologous end joining mechanisms. As similar to mature chondrocytes, irradiated hiPSC-DCH cells revealed accumulation of cells in G2 phase. Overall, the hiPSC-DCH cells were characterized by low levels of ROS, cPARP and high levels of senescence.

**Conclusions:**

The chondrocyte-like cells derived from hiPSC demonstrated features characteristic of both mature chondrocytes and “parental” hiPSCs. The main difference between hiPSC-derived chondrocytes and hiPSCs and mature chondrocytes appears to be the more efficient DDR mechanism of hiPSC-DCHs. The unique properties of these cells suggest that they could potentially be used safely in regenerative medicine if these preliminary findings are confirmed in future studies.

## Introduction

Stem cells (SCs) are a highly promising approach in regenerative medicine. However, their use is not without risk given that the response of SCs and SC-derived components to ionizing radiation (IR) treatment is poorly understood [1]. Although human embryonic stem cells (hESCs) and human induced pluripotent stem cells (hiPSCs) present similar DNA damage response (DDR) mechanisms, including cell cycle arrest in G_2_/M phases and efficient DNA repair, hiPSCs seems more prone to genomic instability, which is strongly associated with the reprogramming process and prolonged culture [2]. As a result, hiPSCs often develop trisomy 12 and 8, amplification of 20q11.21, and unique copy number variations (CNVs) [2]. Epigenetic differences between hESCs and hiPSCs also play an important role in their respective tumorigenicity. The reprogramming process is often associated with epigenetic alterations and epigenetic “memory” may also lead to tumorigenicity in hiPSCs and, consequently, in their derivatives [3].

Several different strategies can be considered to decrease the risk of tumorigenicity of hiPSC- based components, including terminal differentiation, elimination of residual pluripotent SCs, and interference with tumor-associated genes [4]. Hiura et al. (2013) investigated imprinting status and expression levels of eight imprinted genes and the methylation status of their differentially methylated regions in five hiPSCs cell lines. Those authors found that hiPSCs can exhibit loss of imprinting (LOI), which is present not only at early passages, but maintained during long-term culture. This finding is relevant given that LOI appear in many types of malignant tumors, and thus the presence of LOI may decrease the potential utility of hiPSCs in clinical applications [5]. In addition, the “open” chromatin configuration in hiPSCs leads to pluripotency selection in the cell population, which influences the intrinsic instability of these cells [6]. For these reasons, selecting the optimal reprogramming method is crucial. HiPSCs obtained through the integrating method have higher maximum sizes and more CNVs in the genomes than those obtained through non-integrating protocols. Furthermore, integrating hiPSCs display more single nucleotide variations and mosaicism [7]. The available evidence (Luo, et al. 2014) indicates that the addition of antioxidants to prolonged culture of hiPSCs modestly decreases the level of intracellular reactive oxygen species (ROS) and does not influence the expression of *53BP1* and *pATM*, which are engaged in DNA damage and repair. Thus, supplementation with low doses of antioxidant cocktails improves the genomic stability of hiPSCs by decreasing DNA damage [8]. All of these factors may influence DDR mechanisms in cells generated from hiPSCs.

SC-derived cells such as chondrocytes are a promising tool in head and neck reconstruction, which remains a complicated and challenging area. These cells can be used as a component of constructs engineered at the cellular and basic tissue levels such as cartilage, bones, esophagus, trachea, vessels, and nerve [9]. However, data on the response of SC-derived chondrocytes to IR and therefore on their potential tumorigenicity are lacking. In this context, we conducted the present study in which we investigated the response of hiPSC-derived chondrocytes (hiPSC-DCHs) treated with IR. Our findings suggest that hiPSC-DCHs share DDR mechanisms of both “parental” hiPSCs and mature chondrocytes: they readily form double strand breaks (DSBs), possess efficient DNA repair mechanisms involving both Homologous Recombination (HR) and Non-homologous End Joining (NHEJ), do not undergo oxidative stress or massive death, and are highly prone to arrest of cells in G2 phase and senescence after IR. This work contributes to improve our understanding of the processes and mechanisms responsible for maintaining genetic stability in hiPSC-DCHs during radiotherapy (RT), and these data may be useful to evaluate the potential safety of these cells in clinical practice.

## Methods and materials

### Cell culture

The commercially available hiPSC cell line ND41658*H (Coriell Cell Repository, NY, USA) was cultured as described elsewhere [10].

### Embryoid body (EB) formation and chondrogenesis *in vitro*

HiPSCs were used to form EBs. After 7 days, the EBs were differentiated into a chondrogenic lineage in a defined medium supplemented with TGF-β (10 ng/ml) according to a previously established protocol [11].

### Irradiation and dosimetry

3 x 10^6^ hiPSCs, hiPSC-DCHs, and mature chondrocytes (HC-402-05a cell line, ECACC) were irradiated at room temperature in the recommended medium using Gammacell 1000 Elite (TeamBest Theratronics, Canada) at 0, 1, 2, and 5 Gy (dose rate- 2.5 Gy/min). Immediately following irradiation, cells were incubated for 1h, 5h, 9h, 24h and 5 days (to evaluate senescence) in a humidified atmosphere of 5% CO_2_ at 37°C before further analyses. Calibration of the irradiation source in Gammacell 1000 Elan (MDS Nordion, Canada) was performed using Gafchromic EBT films (ISP Corporation, Wayne, NJ, USA) and thermoluminescent dosimetry detectors (TLD) as described previously [12]. The signal was measured and calculated using the HARSHAW TLD Model 3500 Manual Reader (ThermoFisher Scientific, MA, USA).

### Flow cytometry analysis of γH2AX, reactive oxygen species (ROS), cell cycle and Cleaved PARP-1

Cells (hiPSCs, hiPSC-DCHs, and mature chondrocytes) were stained for γH2AX with the Alexa Fluor 647 Mouse Anti-H2AX (pS139) (560447, BD Biosciences, NJ, USA) and for cPARP with the PE Mouse Anti-Cleaved PARP (Asp214) antibodies (562253, BD Biosciences, NJ, USA). ROS detection (immediately after IR) was based on CellROX Green Flow Cytometry Assay Kit (C10492, Thermo Fisher Scientific, MA, USA), where tert-butyl hydroperoxide (TBHP) served as a positive control. Cell cycle analysis (9h after IR) was performed with the use of propidium iodide (P1304MP, Thermo Fisher Scientific MA, USA). All procedures was carried out according to the manufacturer’s instructions. Cells were resuspended in 1 ml staining buffer and analyzed with a flow cytometer (BD Accuri C6, NJ, USA). Fluorescence intensity in arbitrary units was plotted in histograms and the mean fluorescence intensity was calculated. Data were analyzed using FlowJo software (FlowJo v10; LLC, Ashland, OR, USA).

### Reverse Transcriptase-PCR and Real-Time PCR

Total RNA was extracted from cells with Direct-zol RNA MiniPrep columns (Zymo Research, CA, USA). One μg of total RNA per 20 μl reaction volume was reverse-transcribed using the iScript cDNA Synthesis Kit (Bio-Rad, CA, USA). Real Time-PCR reactions were performed using the LightCycler 480 Probes Master (Roche, Switzerland) and the appropriate probe for each primer. cDNA samples were analyzed for genes of interest and for the reference gene *GAPDH* (05-190-541-001, Roche Diagnostics, Switzerland). The expression level for each target gene was calculated as -2^ΔΔct^. The reaction was performed in triplicate for the gene of interest. Real-time polymerase chain reaction for individual genes expression analysis was carried out using LightCycler 96 with specific primers (S6 Table) designed with the Universal Probe Library software (Roche Diagnostics, Switzerland).

### Western blot analysis

Total proteins for the Western blot analysis were extracted from the cells (hiPSCs, hiPSC-DCHs, and HC-402-05a) 9h after irradiation. Cells were collected, washed with PBS, and homogenized with RIPA buffer (ThermoFisher Scientific, MA, USA). After centrifugation at 13000 rpm at 4˚C for 30 min, the supernatant was transferred into new tubes. The concentration of the protein sample was measured using the Pierce BCA Protein Assay Kit (23225, MA, USA). Ten micrograms of total protein of each cell extract was resolved by Tris/Glycine/Sodium dodecyl sulphate polyacrylamide gel electrophoresis and transferred to a polyvinylidinedifluoride membrane (1704156, Bio-Rad, CA, USA). Nonspecific binding was blocked by incubation in 5% non-fat milk in Tris-buffered saline and Tween 20 at room temperature for 1h. Blots were then probed overnight at 4˚C with anti-β-actin (N-21: sc-130656 1:250, Santa Cruz, TX, USA), Rad51 1:250 (ab46981, Abcam, UK) and XRCC4 1:500 (ab97351, Abcam, UK). Immunoreactive bands were then probed for 1h at room temperature with the appropriate horseradish peroxidase (HRP)-conjugated secondary anti-Rabbit IgG-HRP 1:2000 (7074S, Cell Signaling Technology, MA, USA). Protein bands were detected by WesternBright Quantum HRP substrate (Advansta, CA, USA) and imaged using a ChemiDoc Imaging Systems (Biorad, CA, USA).

### Senescence analysis

The cells were seeded on 12-well plates and prepared according to the manufacturer's instructions (QIA117, Merck Millipore, Germany). First, the cells were fixed at room temperature for 15 minutes. Next, the cells were rinsed with PBS and stained with Staining Solution Mix (0.5 ml per well) consisting of 470 μl of Staining Solution, 5 μl of Staining Supplement, and 25 μl of 20 mg/ml X-gal in DMF at 37°C overnight. Finally, microscopic analysis was performed (200x total magnification).

### Statistical analysis

All experiments were performed at least 3 times. Results are presented as means ± standard deviation. Comparisons between the study groups and controls were performed using one-way analysis of variance followed by post-hoc analysis using Dunnett’s multiple comparison test. Comparisons between the study groups and controls were performed with GraphPad Prism (version 5.0; GraphPad Software, Inc., La Jolla, CA, USA). P<0.05 was considered to indicate a statistically significant difference.

## Results and discussion

SCs are characterized by unique DDR mechanisms and, consequently, DNA damage-induced adaptations such as anaerobic metabolism, fewer mitochondria, and most importantly, highly efficient DNA repair capacity that decreases during the differentiation process [13].

Genetic variations in hiPSCs can occur through three different mechanism. First, pre-existing variations in parental somatic cells can be intensified by the cloning procedure during hiPSC generation. Second, reprogramming-induced mutations can occur during the reprogramming process. Third, passage-induced mutations can be acquired during prolonged culture [14]. Liu et al. [15] found that iPSCs develop genetic instability during prolonged cell culture. In their study, which was performed in iPSCs derived from pig tissues (considered comparable to hiPSCs), the authors found that the DNA repair capacity of the cells decreased as the number of passages increased. This decreased DNA repair capacity was linked to reduced pluripotency and differentiation capacity, accumulation of DNA damage, and failed apoptosis. According to the study's authors, the resulting genetic instability (and the decreased DNA repair capacity) are probably caused by an incomplete reprogramming process [15]. Those studies emphasize the question surrounding the genetic stability and potential tumorigenicity of SC-derived cells, particularly those generated from hiPSCs with genetic instability.

Osteo- and chondrogenic tumors of the axial skeleton can be divided into three groups according to the histologic diagnosis and biological behavior: 1) chordomas and chondrosarcomas, 2) osteogenic sarcomas, and 3) giant cell tumors, and osteo- and chondroblastomas [16]. Tissue engineering involving autologous chondrocytes or SCs is a promising strategy for cartilage reconstruction, particularly in the head and neck region [17]. For this reason, hiPSC-DCHs will be inevitably be exposed to IR in the near future, both during both diagnosis (e.g., computed tomography) and treatment (radiotherapy) that, in turn, potentially may lead to development of aforementioned chondrogenic tumors.

A major concern regarding the use of hiPSC-DCHs for regenerative purposes in humans is that the response of such cells to IR remains poorly understood. Therefore, the aim of this study was to examine major DDR mechanisms of hiPSC-DCHs treated with IR and to compare those results with those obtained from “parental” hiPSCs and mature chondrocytes (HC-402-05a cell line), which were used as controls.

**Fig 1:**
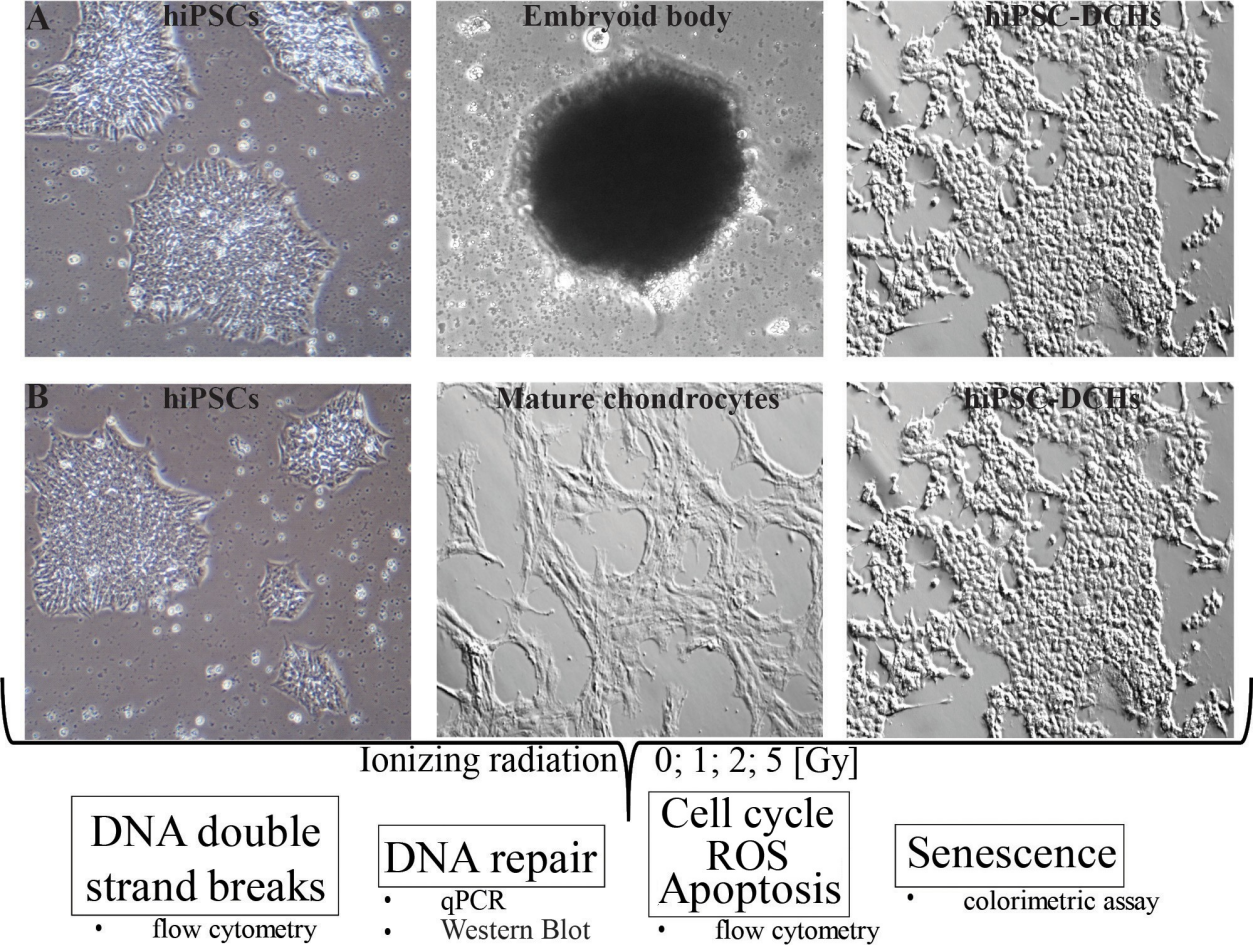
Schematic of the experimental design. First, human induced pluripotent stem cells (hiPSCs) were differentiated into a chondrogenic lineage (hiPSC-DCHs) *via* embryoid bodies consisting of three primary germ layers (endo-, meso- and ectoderm) in a medium supplemented with TGF-β3 (10 ng/ml) as a GF with the most chondrogenic potential (A). Then the hiPSC-DCHs were treated with ionizing radiation (0; 1; 2; 5 [Gy]) and the following response characteristics were analyzed: kinetics of DNA double strand breaks, DNA repair mechanisms at the gene and protein level, markers of apoptosis and senescence. HiPSCs and mature chondrocytes served as controls (B).

**Fig 2:**
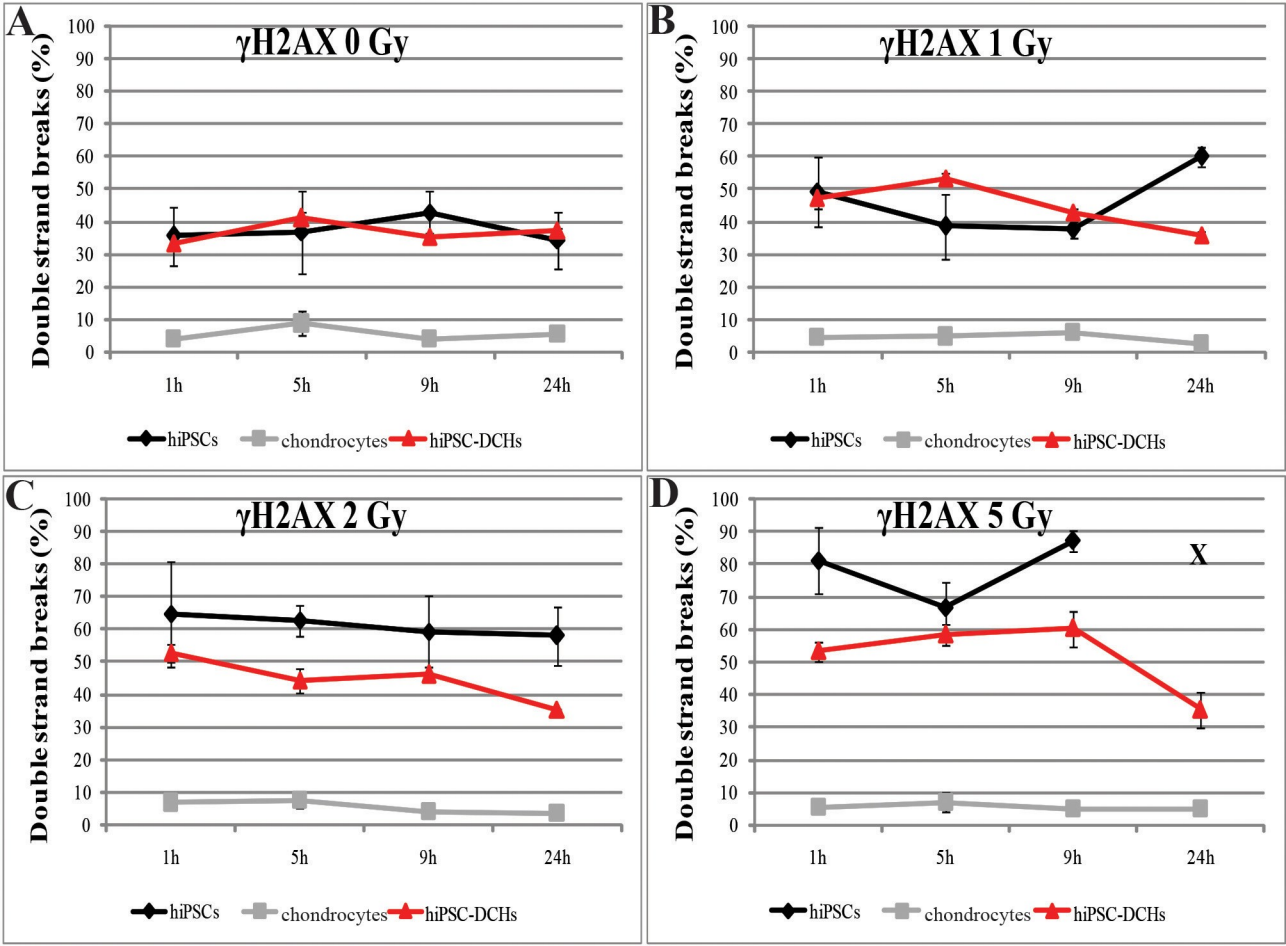
Formation of DNA double strand breaks (determined by γH2AX staining) were evaluated by flow cytometry analysis at 1, 5, 9 and 24h after irradiation. Human induced pluripotent stem cell (hiPSC)-derived chondrocytes (hiPSC-DCHs) obtained after chondrogenic differentiation *in vitro* of hiPSCs were irradiated at the following doses: 0 (A); 1 (B); 2 (C); and 5 (D) [Gy] and compared to hiPSCs and mature chondrocytes (HC-402-05a cell line) which received the same doses. HiPSCs treated with 5 Gy underwent massive cell death 24h after IR exposure (X).

First, we performed *in vitro* chondrogenesis of hiPSCs according to an established protocol to obtain chondrogenic-like cells [11] (Fig 1A; S1 Fig). Then, these hiPSC-DCHs were irradiated and analyzed (Fig 1B). We found that hiPSCs and hiPSC-DCHs presented a similar number of DNA DSBs: both of these cell types readily developed DNA DSBs and both were susceptible to DNA damage during IR. However, unlike the hiPSCs, the hiPSC-DCHs were characterized by highly efficient DNA repair mechanisms. The decrease in the number of DNA DSBs was particularly noticeable at 9h post-IR (Fig 2A, 2B, 2C and 2D), a finding that contrasts markedly with the hiPSCs, which did not demonstrate DNA DSB repair and which underwent massive cell death after 5 Gy of IR (Fig 2D). Mature chondrocytes, by contrast, showed no significant changes in the formation of DNA DSBs after IR: the level of γH2AX was comparable in both untreated and IR-treated mature chondrocytes (Fig 2A, 2B, 2C and 2D; S1 Table).

**Fig 3:**
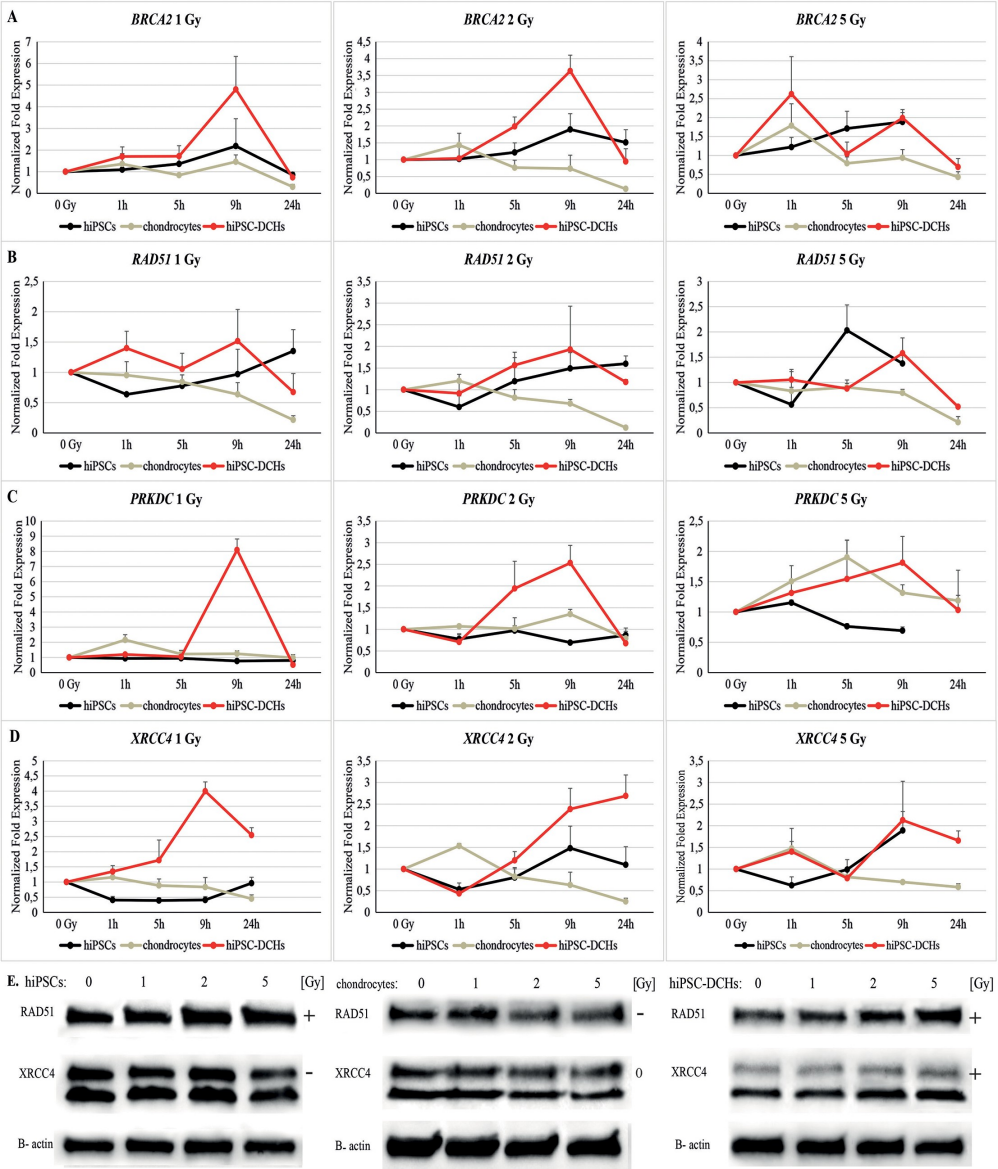
Activation of DNA repair mechanisms for DNA double strand breaks (1, 5, 9, 24h after irradiation) in irradiated hiPSC-derived chondrocytes (hiPSC-DCHs) was assessed by quantitative PCR. Expression of the *BRCA2* and *RAD51* genes (homologous recombination) (A and B) and *PRKDC* and *XRCC4* (non-homologous end-joining) (C and D) was greater in irradiated hiPSC-DCHs compared to controls (i.e., hiPSCs and chondrocytes). These results were confirmed at the protein level by Western Blot analysis (9h after irradiation) (E).

HiPSC-DCHs strongly activate DNA repair mechanisms when exposed to IR. The *BRCA2* gene (HR mechanism) was highly expressed in these cells. A similar trend was observed in “parental” hiPSCs. Nevertheless, *BRCA2* expression was lower in the hiPSCs than in the hiPSC-DCHs. By contrast, mature chondrocytes showed decreased or unchanged *BRCA2* expression levels (Fig 3A; S2 Table).

*RAD51* gene expression (HR mechanism) was higher in both hiPSCs and hiPSC-DCHs to mature chondrocytes. However, this gene was activated more effectively by hiPSC-DCHs than by hiPSCs. As in the case of *BRCA2* expression, *RAD51* expression was lower in HC-402-05a cells than in the other analyzed cells (Fig 3B; S3 Table).

The *PRKDC* gene (NHEJ mechanism) was highly expressed in both hiPSC-DCHs and mature chondrocytes. However, this gene was more highly expressed in the hiPSC-DCHs than in hiPSCs. The difference between *PRKDC* expression in these two types of cells decreased as the radiation dose increased. By contrast, hiPSCs did not present increased levels of *PRKDC* expression (Fig 3C; S4 Table).

*XRCC4* gene expression (NHEJ mechanism) was highest in the hiPSC-DCHs. However, both hiPSCs and hiPSC-DCHs showed increased expression of this gene, whereas *XRCC4* expression decreased in mature chondrocytes (Fig 3D; S5 Table).

**Fig 4:**
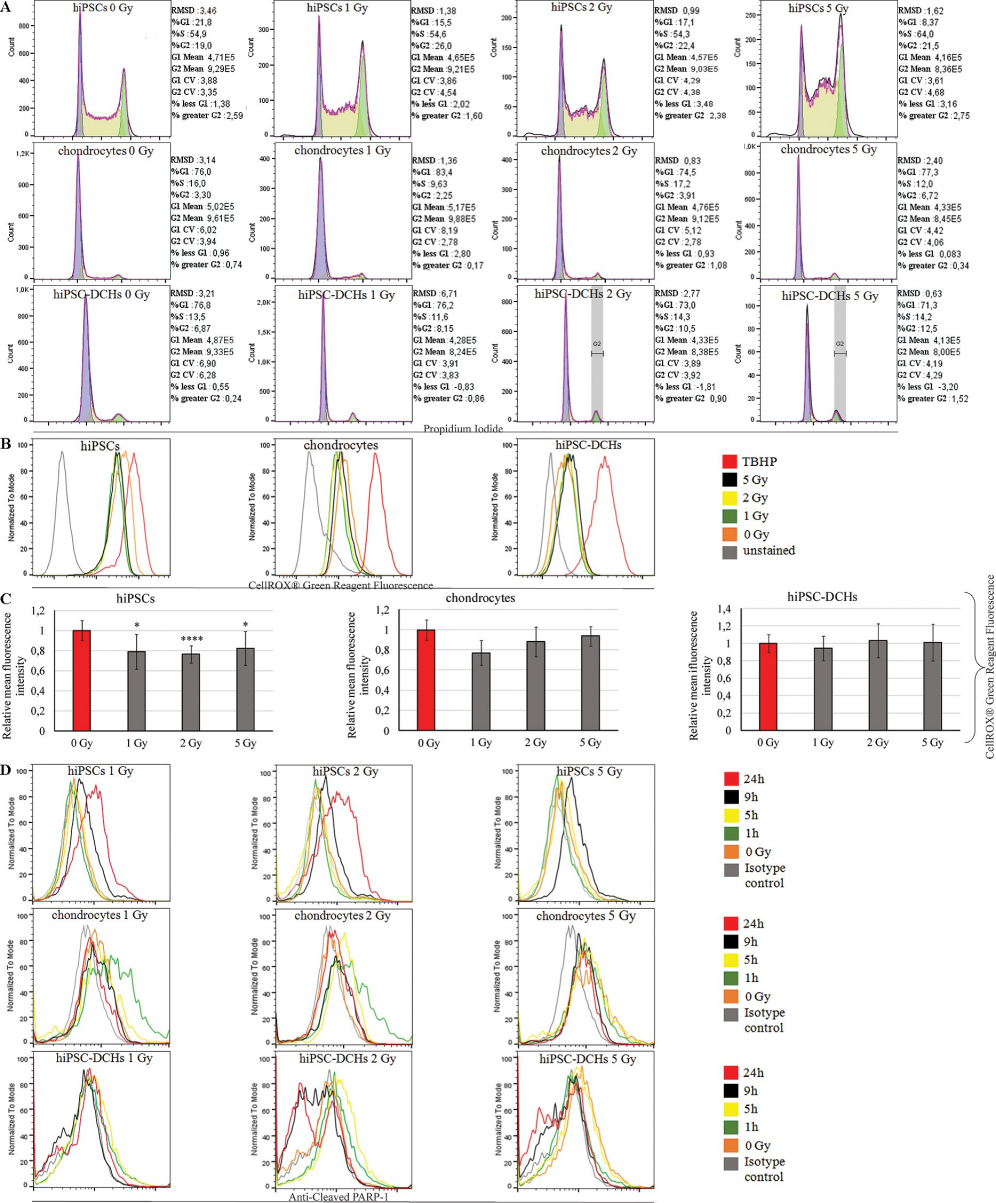
Based on flow cytometry analysis with the propidium iodide the irradiated (9h after IR) hiPSC-DCHs and mature chondrocytes pointed at accumulation and cell cycle arrest in G2 phase. In turn, hiPSCs exposed to IR demonstrated high level of cells found in S phase (A). All investigated cells (hiPSCs, hiPSC-DCHs and chondrocytes) showed little differences in ROS level measured–using CellROX Green Reagent and tert-butyl hydroperoxide as a positive control- immediately after IR. HIPSCs are characterized by both initial and IR-caused high level of ROS (B and C). HiPSCs treated with ionizing radiation (IR) presented high levels of apoptosis (as a cleaved PARP-1 staining), with massive cell death occurring 24h after high dose IR. Mature chondrocytes (HC-402-05a cell line) exposed to IR had lower levels of apoptosis than hiPSCs. The level of hiPSC-derived chondrocytes (hiPSC-DCHs) undergoing apoptosis at 1, 5, 9, and 24h after IR was comparable to that observed in mature chondrocytes. Apoptosis levels in irradiated hiPSC-DCHs decreased within 24h (D).

The irradiated hiPSCs demonstrated accumulation of cells in S phase (Fig 4A). On the contrary, both mature chondrocytes and hiPSC-DCHs revealed arrest of cell cycle in G2 phase 9h after IR (as a most sensitive point that we selected; Fig 4A). Moreover, chondrocyte-like cells obtained from hiPSCs revealed characterization of cell cycle similar to fully differentiated cells (hiPSC-DCHs vs chondrocytes). In that case the majority of cells were noticeable in G1 phase. In contrast, non-irradiated hiPSCs as highly proliferative type of cells demonstrate the highest percentage of S phase (Fig 4A).

The IR did not cause a dramatic differences in the ROS level in all investigated types of cells (hiPSCs, hiPSC-DCHs and mature chondrocytes) (Fig 4B and 4C). However, there was a significant dissimilarity between overall profile of hiPSCs and differentiated cells (both hiPSC-DCHs and chondrocytes). The non- and irradiated hiPSCs showed the relatively high level of ROS- that was shifted to the positive control- in contrast to differentiated cells (Fig 4B).

**Fig 5:**
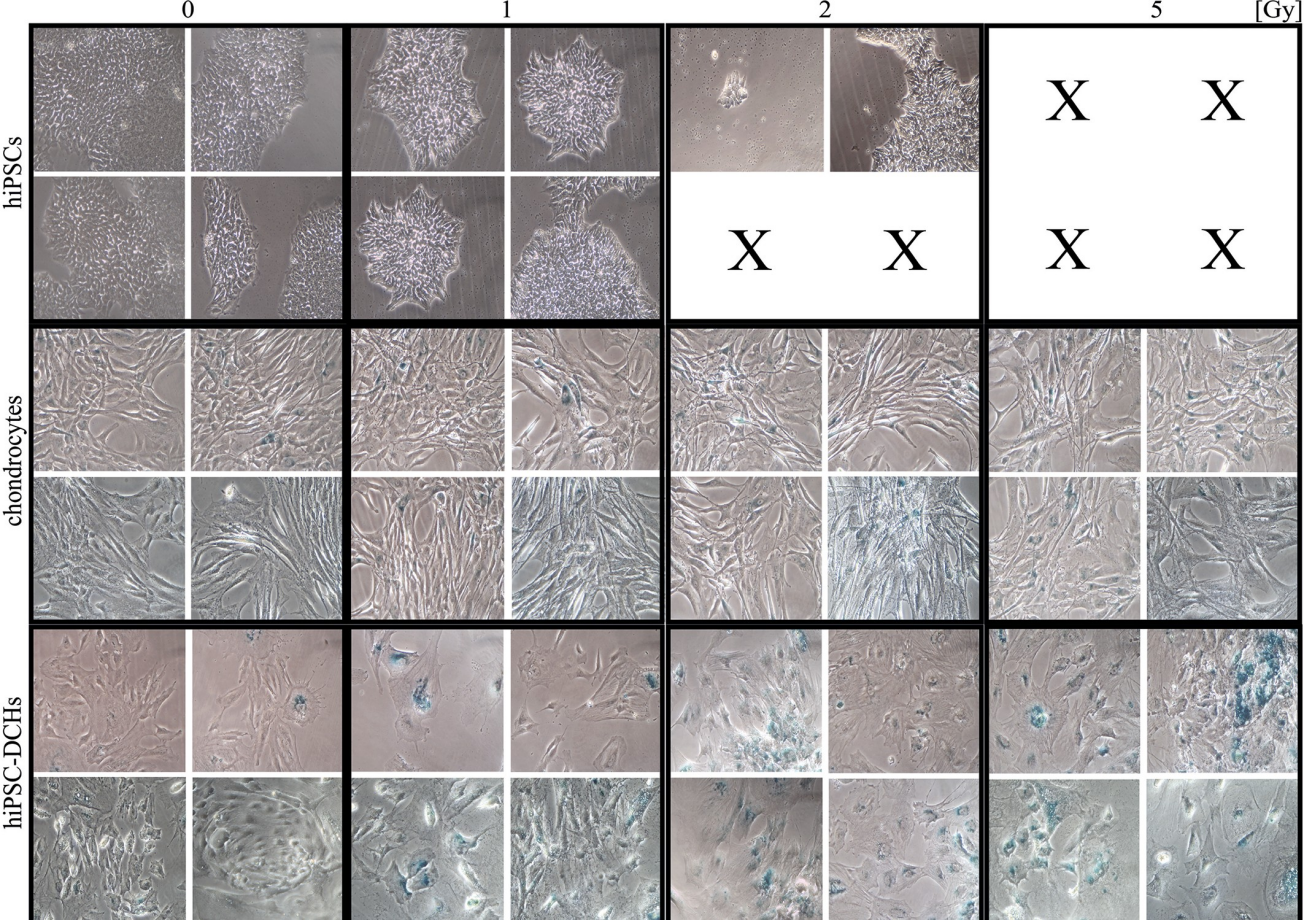
The number of cells undergoing senescence (with higher level of lysosomal enzyme β-Galactosidase) after ionizing radiation (IR) (0; 1; 2; 5 [Gy]) was significantly higher in hiPSC-derived chondrocytes (hiPSC-DCHs) than in mature chondrocytes (HC-402-05a cell line) and (especially) in hiPSCs. The analysis was performed 5 days after IR exposure. Massive cell death (X) was observed in hiPSCs treated with 2 and 5 Gy doses of IR.

The increased levels of apoptosis after IR show that hiPSCs were highly susceptible to the effects of radiation (Fig 4D). Since hiPSCs underwent massive cell death 24h after 5 Gy of IR, it was not feasible to analyze apoptosis at this time point. By contrast, chondrocytes (both hiPSC-DCHs and mature chondrocytes) did not undergo easily apoptosis after IR (Fig 4D): at 24h post-IR, cPARP levels in the chondrocytes had returned to pre-treatment levels. Although hiPSC-DCHs do not easily undergo apoptosis, they are prone to senescence, seen as blue cells (with β-galactosidase activity) at 5 days post-IR (Fig 5). Mature chondrocytes were characterized by individual positive cells. Finally, senescence as a cell death mechanism was uncommon in hiPSCs because of high rates of apoptosis after IR.

One study, which evaluated rabbit chondrocytes exposed to IR, showed that components of the cartilaginous matrix such as glycosaminoglycan (GAG) are relatively radioresistant [18]. However, DNA synthesis was substantially suppressed and there was no increase in the number of cultured chondrocytes after IR. A primary rat costochondral growth cell culture model was used to demonstrate the effects of IR on proliferative chondrocytes [19]. That study found that the response of the irradiated chondrocytes was dose-dependent: as the dose increased, proliferation decreased, while cytotoxity, several markers of apoptosis, and radiation-induced cellular differentiation all increased; in addition, cell synthetic activity was disrupted. These effects are strongly correlated with the parathyroid hormone-related protein-Indian hedgehog proliferation-maturation pathway [19]. Another study [20] showed that primary rabbit articular chondrocytes undergo senescence in response to IR: chondrocytes that presented senescence demonstrated significantly decreased regenerative capacity, permanent cell cycle arrest, and a large, flat morphology. In the irradiated chondrocytes, the ERK and p38 mitogen-activated protein kinase pathways were activated. Interestingly, low-dose radiation (LDR) up to 2 cGy had a beneficial effect on cartilage. LDR inhibits interleukin (IL)-1β- induced chondrocyte destruction, dedifferentiation, and inflammation processes through the arrest of catenin signaling, apparently without causing any side effects such as apoptosis and senescence [21]. Osteochondral allografts used as biological implants in the reconstruction of post-traumatic cartilage defects, osteoarthritis, osteonecrosis, osteochondritis dissecans, and tumour resection often lead to immune reaction characterized by pannus formation. This immune response can be mediated with RT: low-dose fractionated RT induces immune suppression without side effects. By contrast, although single-fraction high dose RT also causes immune suppression, it adversely affects chondrocytes, thus leading to permanent cartilage defects [22]. Hamdi and colleagues (2016) pointed out that the biologic effect exerted by C-ion beam—routinely used to treat chondrosarcoma—measured in 2D human articular chondrocyte cell culture might be overestimated compared to the clinical reality. The 3D cartilage model (3DCaM) provides more accurate results: in 3DCaM, higher linear energy transfer does not induce more senescence compared to X-rays, in contrast to the results obtained with 2D cell culture [23]. However, no data are available regarding the response of irradiated chondrocytes derived from hiPSCs.

NHEJ is active during the cell cycle and its activity increases as cells progress from G1 to G2/M cell cycle phases. HR in somatic cells is mainly absent in G1, most active in S, and declines in G2/M phases. The overall efficiency of NHEJ is higher than HR at all cell cycle stages. In conclusion, human somatic cells utilize error-prone NHEJ as the major DSB repair pathway at all cell cycle stages, while HR is used, mainly, in the S phase [24]. Mao et al. (2008) demonstrated that NHEJ is a faster and more efficient DSB repair pathway than HR. They showed that NHEJ of compatible ends (NHEJ-C) and NHEJ of incompatible ends (NHEJ-I) are quick processes, which can be completed in approximately 30 min. In turn, HR is much slower and takes at least 7h to complete. The authors concluded that in proliferating cells NHEJ repairs 75% of DSBs while HR repairs the remaining 25%. They also suggest that an overall 3:1 ratio between NHEJ and HR may be a general phenomenon for mammalian cells. It should be noted that the observed frequencies refer to actively proliferating cells. In G1-arrested quiescent or differentiated cells the frequency of HR is likely to be much lower. In mammals NHEJ is the preferred pathway [25]. The choice may be conditioned by genome composition. In large repetitive genomes of animals overly efficient HR may result in deleterious genomic rearrangements, such that NHEJ may be a safer and more reliable choice [25,26]. Schneider and co-workers (2012) showed that terminally differentiated astrocytes are–in contrast to the parental neural stem cell (SC)—radioresistant and do not undergo apoptosis upon irradiation. Nevertheless, despite suppressed DDR signaling pathways, DNA damage induced phosphorylation of H2AX at S139 is still clearly detectable in astrocytes [27].

SC differentiation induced by treatment with NO donor (NOC-18) has no effect on DSB repair by NHEJ but notably reduced DSB repair by HR. Those studies suggest that DNA repair by HR is impaired in differentiated cells. Consequently, Differentiated cells have a reduced frequency of foci formation by RAD51, BRCA1, and other HR-related product. Those findings support studies indicating that both ESCs and iPSCs repair DNA lesions by HR compared with their differentiated derivatives. Some studies have also shown that, compared with neural SCs, terminally differentiated descendant astrocytes lack functional DDR signaling. Mature astrocytes and dopaminergic neurons exhibit significantly higher residual damage, in comparison with their undifferentiated and neuronal progenitor cells, as demonstrated by the delayed disappearance of γ-H2AX foci 8–12 hr post irradiation [28].

Venkatesh and collaborators (2016) showed that considerably higher numbers of DSBs are formed in pluripotent cells, compared to differentiated cells, in response to high doses of IR. At the same time, the number of repair centers formed was comparable between various pluripotent SC cell lines. They found that heterochromatin in hESC is confined to distinct regions; whereas in differentiated cells it is distributed more evenly within the nuclei. Using the comet assay they showed that the same dose of IR led to considerably more DSBs in hESC than in their differentiated derivatives, normal human fibroblasts, and a cancer cell line. Those authors also found that in hESC, DNA repair foci localized almost exclusively outside the heterochromatin regions [29].

DNA damage induces cell cycle arrest and DNA repair or apoptosis in proliferating cells. Terminally differentiated cells are permanently withdrawn from the cell cycle and partly resistant to apoptosis. As reported by Latella et al. (2004) radioresistance in myotubes might reflect a differentiation-associated, pathway- selective blockade of DNA damage signaling downstream of ATM. This mechanism appears to preserve IR-induced activation of the ATM-H2AX-MRE11/Rad50/Nbs1 lesion processing and repair pathway yet restrain ATM-p53-mediated apoptosis, thereby contributing to life-long maintenance of differentiated muscle tissues.

In those study, model of skeletal muscle differentiation to investigate the responses of undifferentiated versus terminally differentiated cells to genotoxic agents was used. A comparative analysis of the responses to IR in muscle cells both before and after terminal differentiation was performed. The analysis revealed that the IR-activated pathway is interrupted in myotubes at the level of Ser15(h)/18(m) phosphorylation of p53, leading to the acquisition of an apoptosis-resistant phenotype upon IR exposure [30].

Despite the numerous studies described above, until now there has been a notable lack of data on inducing HR and NHEJ mechanisms in hiPSC-DCHs treated with IR. The obtained results clearly indicate that hiPSC-DCHs readily activate members of both NHEJ and HR. The participation of both processes is extremely high in hiPSC-DCHs. Importantly, although hiPSC-DCHs strongly activate DDR mechanisms–including error-prone NHEJ—data on proper carcinogenesis of these cells are lacking. Moreover, since literature data pointed out that HR participates in DSBs repair formed in SCs to a large extent, we can assume that hiPSC-DCHs although unmistakable characteristics of mature chondrocytes, they also demonstrate features of parental SCs’ DDR processes.

## Conclusion

Chondrocytes differentiated from hiPSCs are a highly promising tool in tissue engineering, especially for reconstruction of the head and neck area. However, “parental” hiPSCs may present genetic instability and thus chondrocytes derived from such hiPSCs after *in vitro* differentiation may also present altered DDR mechanisms, which may result in tumorigenesis. The present study was performed to improve our understanding of DDR response in SC-derived cells. The main finding of this study is that although hiPSC-DCH cells readily form DSBs and accumulate in G2 phase, they also are characterized by highly efficient DNA repair mechanisms. Moreover, hiPSC-DCHs appear to be more likely to undergo senescence rather than apoptosis after IR exposure. This finding shows that the differentiation process has an important impact on the DDR mechanisms of irradiated cells. Cells derived from hiPSCs possess properties of the “parental” hiPSCs as well as those of fully mature, differentiated chondrocytes. More data are needed to better elucidate the key aspects of DDR mechanisms activated in hiPSC-derived chondrocytes exposed to IR.

## Supporting information

S1 Fig Chondrocyte-like cells were obtained from hiPSCs (hiPSC-DCHs) according to a previously-established and published protocol [11]. HiPSC-DCHs demonstrated the presence of *inter alia* type II collagen (COLL II), cartilage oligomeric matrix protein (COMP), and aggrecan (AGG).
(TIF)

S1 Table The statistical analyses of γH2AX, formation in analyzed cells using the unpaired one-way analysis of variance (ANOVA). Results are expressed as mean ± standard deviation *P<0.05, ** *P <* 0.01, *** *P <* 0.001, **** P<0.0001 compared with control- hiPSCs cell line.
(DOCX)

S2 Table The statistical analysis of *BRCA2* expression formation in analyzed cells using the unpaired one-way analysis of variance (ANOVA) (A,B).
A
Results are expressed as mean ± standard deviation *P<0.05, ** *P* < 0.01, *** *P* < 0.001, **** P<0.0001 compared with control- HC-402-05a cell line.
B
Results are expressed as mean ± standard deviation *P<0.05, ** *P* < 0.01, *** *P* < 0.001, **** P<0.0001 compared with control- hiPSCs cell line.
(DOCX)

S3 Table The statistical analysis of *RAD51* expression formation in analyzed cells using the unpaired one-way analysis of variance (ANOVA) (A,B).
A
Results are expressed as mean ± standard deviation *P<0.05, ** *P* < 0.01, *** *P* < 0.001, **** P<0.0001 compared with control- HC-402-05a cell line.
B
Results are expressed as mean ± standard deviation *P<0.05, ** *P* < 0.01, *** *P* < 0.001, **** P<0.0001 compared with control- hiPSCs cell line.
(DOCX)

S4 Table The statistical analysis of *PRKDC* expression formation in analyzed cells using the unpaired one-way analysis of variance (ANOVA) (A,B).
A
Results are expressed as mean ± standard deviation *P<0.05, ** *P* < 0.01, *** *P* < 0.001, **** P<0.0001 compared with control- HC-402-05a cell line.
B
Results are expressed as mean ± standard deviation *P<0.05, ** *P* < 0.01, *** *P* < 0.001, **** P<0.0001 compared with control- hiPSCs cell line.
(DOCX)

S5 Table The statistical analysis of *XRCC4* expression formation in analyzed cells using the unpaired one-way analysis of variance (ANOVA) (A,B).
A
Results are expressed as mean ± standard deviation *P<0.05, ** *P* < 0.01, *** *P* < 0.001, **** P<0.0001 compared with control- HC-402-05a cell line.
B
Results are expressed as mean ± standard deviation *P<0.05, ** *P* < 0.01, *** *P* < 0.001, **** P<0.0001 compared with control- hiPSCs cell line.
(DOCX)

S6 Table Forward and reverse primer sequences. Abbreviations: BRCA2 indicates breast cancer 2; RAD51, RAD51 recombinase; PRKDC, DNA-dependent protein kinase catalytic subunit; XRCC4, X-ray repair complementing defective repair in Chinese hamster cells 4; and PRKDC, DNA-dependent protein kinase catalytic subunit.
(DOCX)

## References

[1] Suchorska WM, Augustyniak E, Łukjanow M. Genetic stability of pluripotent stem cells during anti-cancer therapie. Exp Ther Med. 2016; 11(3):695–702. 10.3892/etm.2016.2993 26997981 PMC4774348

[2] Martins-Taylor K, Xu RH. Concise Review: Genomic Stability of Human Induced Pluripotent Stem Cells. Stem Cells. 2012; 30(1):22–7. 10.1002/stem.705 21823210

[3] Garitaonandia I, Amir H, Boscolo FS, Wambua GK, Schultheisz HL, Sabatini K, et al Increased risk of genetic and epigenetic instability in human embryonic stem cells associated with specific culture conditions. PLoS One. 2015; 10(2):e0118307 10.1371/journal.pone.0118307 25714340 PMC4340884

[4] Ben-David U, Benvenisty N. The tumorigenicity of human embryonic and induced pluripotent stem cells. Nat Rev Cancer. 2011; 11(4):268–77. 10.1038/nrc3034 21390058

[5] Hiura H, Toyoda M, Okae H, Sakurai M, Miyauchi N, Sato A, et al Stability of genomic imprinting in human induced pluripotent stem cells. BMC Genet. 2013; 14:32 10.1186/1471-2156-14-32 23631808 PMC3751563

[6] Blasco MA, Serrano M, Fernandez-Capetillo O. Genomic instability in iPS: time for a break. EMBO J. 2011; 30(6):991–3. 10.1038/emboj.2011.50 21407252 PMC3061043

[7] Kang X, Yu Q, Huang Y, Song B, Chen Y, Gao X, et al Effects of Integrating and Non-Integrating Reprogramming Methods on Copy Number Variation and Genomic Stability of Human Induced Pluripotent Stem Cells. PLoS One. 2015; 10(7):e0131128 10.1371/journal.pone.0131128 26131765 PMC4488894

[8] Luo L, Kawakatsu M, Guo CW, Urata Y, Huang WJ, Ali H, et al Effects of antioxidants on the quality and genomic stability of induced pluripotent stem cells. Sci Rep. 2014; 4:3779 10.1038/srep03779 24445363 PMC3896906

[9] Barczak W, Golusiński P, Luczewski L, Suchorska WM, Masternak MM, Golusiński W. The importance of stem cell engineering in head and neck oncology. Biotechnol Lett. 2016; 38(10):1665–72. 10.1007/s10529-016-2163-7 27341837 PMC5010595

[10] Lach MS, Wroblewska JP, Augustyniak E, Kulcenty K, Suchorska WM. A feeder- and xeno-free human induced pluripotent stem cell line obtained from primary human dermal fibroblasts with epigenetic repression of reprogramming factors expression: GPCi001-A. Stem Cell Research. 2017; 4(20):34–37. 10.1016/j.scr.2017.02.004 28395738

[11] Suchorska WM, Augustyniak E, Richter M, Trzeciak T. Comparison of Four Protocols to Generate Chondrocyte-Like Cells from Human Induced Pluripotent Stem Cells (hiPSCs). Stem Cell Rev. 2016; 13(2):299–308. 10.1007/s12015-016-9708-y PMC5380716 27987073

[12] Vandana S, Shaiju VS, Sharma SD, Mhatre S, Shinde S, Chourasiya G, et al Dosimetry of gamma chamber blood irradiator using Gafchromic EBT film. Appl Radiat Isot. 2011; 69(1):130–5. 10.1016/j.apradiso.2010.08.018 20850330

[13] Rocha CR, Lerner LK, Okamoto OK, Marchetto MC, Menck CF. The role of DNA repair in the pluripotency and differentiation of human stem cells. Mutat Res. 2013 752(1):25–35. 10.1016/j.mrrev.2012.09.001 23010441

[14] Yoshihara M, Hayashizaki Y, Murakawa Y. Genomic Instability of iPSCs: Challenges Towards Their Clinical Applications. Stem Cell Rev. 2017; 13(1):7–16. 10.1007/s12015-016-9680-6 27592701 PMC5346115

[15] Liu K, Mao J, Song L, Fan A, Zhang S, Wang J, et al DNA repair and replication links to pluripotency and differentiation capacity of pig iPS cells. PLoS One. 2017; 12(3):e0173047 10.1371/journal.pone.0173047 28253351 PMC5333863

[16] Hug EB, Fitzek MM, Liebsch NJ, Munzenrider JE. Locally challenging osteo- and chondrogenic tumors of the axial skeleton: results of combined proton and photon radiation therapy using three-dimensional treatment planning. Int J Radiat Oncol Biol Phys. 1995 31(3):467–76. 10.1016/0360-3016(94)00390-7 7852108

[17] Zhou L, Ding R, Li B, Han H, Wang H, Wang G, et al Cartilage engineering using chondrocyte cell sheets and its application in reconstruction of microtia. Int J Clin Exp Pathol. 2015; 8(1):73–80. 25755694 PMC4348915

[18] Matsumoto T, Iwasaki K, Sugihara H. Effects of Radiation on Chondrocytes in Culture. Bone. 1994; 15(1):97–100. 8024859 10.1016/8756-3282(94)90898-2

[19] Margulies BS, Horton JA, Wang Y, Damron TA, Allen MJ. Effects of radiation therapy on chondrocytes in vitro. Calcif Tissue Int. 2006; 78(5):302–13. 10.1007/s00223-005-0135-3 16691495

[20] Hong EH, Lee SJ, Kim JS, Lee KH, Um HD, Kim JH, et al Ionizing radiation induces cellular senescence of articular chondrocytes via negative regulation of SIRT1 by p38 kinase. J Biol Chem. 2010 285(2):1283–95. 10.1074/jbc.M109.058628 19887452 PMC2801256

[21] Hong EH, Song JY, Lee SJ, Park IC, Um HD, Park JK, et al Low-Dose γ-Radiation Inhibits IL-1β- Induced Dedifferentiation and Inflammation of Articular Chondrocytes via Blockage of Catenin Signaling. IUBMB Life. 2014; 66(2):128–37. 10.1002/iub.1248 24604706 PMC4321059

[22] Gönç U, Çetinkaya M, Atabek M. The effects of low-dose radiotherapy on fresh osteochondral allografts: An experimental study in rabbits. Acta Orthop Traumatol Turc. 2016; 50(5):572–577. 10.1016/j.aott.2016.08.004 27863947 PMC6197546

[23] Hamdi DH, Chevalier F, Groetz JE, Durantel F, Thuret JY, Mann C, et al Comparable Senescence Induction of Three-dimensional Human Cartilage Model by Exposure to Therapeutic Doses of X-rays or C-ions. Int J Radiat Oncol Biol Phys. 2016; 95(1):139–46. 10.1016/j.ijrobp.2016.02.014 27084635

[24] Mao Z, Bozzella M, Seluanov A, Gorbunova V. DNA repair by nonhomologous end joining and homologous recombination during cell cycle in human cells. Cell Cycle. 2008; 7(18):2902–6. 10.4161/cc.7.18.6679 18769152 PMC2754209

[25] Mao Z, Bozzella M, Seluanov A, Gorbunova V. Comparison of nonhomologous end joining recombination in human cells. DNA Repair (Amst). 2008; 7(10):1765–71. 18675941 10.1016/j.dnarep.2008.06.018 PMC2695993

[26] Vitale I, Manic G, De Maria R, Kroemer G, Galluzzi L. DNA Damage in Stem Cells. 2017; 66(3):306–319. 10.1016/j.molcel.2017.04.006 28475867

[27] Schneider L, Fumagalli M, d’Adda di Fagagna F. Terminally differentiated astrocytes lack DNA damage response signaling and are radioresistant but retain DNA repair proficiency. Cell Death Differ. 2012; 19(4):582–91. 10.1038/cdd.2011.129 21979466 PMC3307974

[28] Mujoo L, Pandita RK, Tiwari A, Charaka V, Chakraborty S, Singh DK, et al Differentiation of Human Induced Pluripotent or Embryonic Stem Cells Decreases the DNA Damage Repair by Homologous Recombination. Stem Cell Reports. 2017; 9(5):1660–1674. 10.1016/j.stemcr.2017.10.002 29103969 PMC5831054

[29] Venkatesh P, Panyutin IV, Remeeva E, Naumann RD, Panyutin UG. Effect of Chromatin Structure on the Extent and Distribution of DNA Double Strand Breaks Produced by Ionizing Radiation; Comparative Study of hESCs and Differentiated Cells Lines. Int J Mol Sci. 2016; 17(1). pii: E58. 10.3390/ijms17010058 PMC4730303 26729112

[30] Latella L, Lukas J, Simone C, Puri P, Bartek J. Differentiation-induced radioresistance in muscle cells. Mol Cell Biol. 2004; 24(14):6350–61. 10.1128/MCB.24.14.6350-6361.2004 15226436 PMC434249

