# Supplementary figures and images for: Chondrocytes differentiated from human induced pluripotent stem cells: Response to ionizing radiation
Source: PLoS One. 2018 Oct 23;13(10):e0205691. doi: 10.1371/journal.pone.0205691 (PMC6198947; doi:10.1371/journal.pone.0205691)

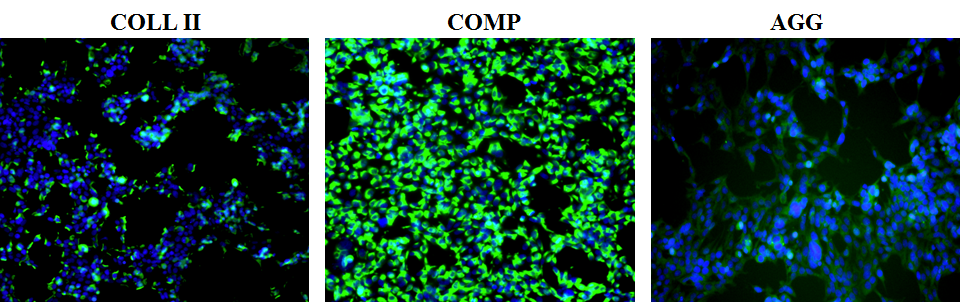

Supplement: S1 Fig — HiPSC-DCHs demonstrated the presence of inter alia type II collagen (COLL II), cartilage oligomeric matrix protein (COMP), and aggrecan (AGG). (TIF) [file pone.0205691.s001.tif]
