# Supplementary material for: Chondrocytes differentiated from human induced pluripotent stem cells: Response to ionizing radiation
Source: PLoS One. 2018 Oct 23;13(10):e0205691. doi: 10.1371/journal.pone.0205691 (PMC6198947; doi:10.1371/journal.pone.0205691)
Supplement: S2 Table — The statistical analysis of BRCA2 expression formation in analyzed cells using the unpaired one-way analysis of variance (ANOVA) (A,B). A Results are expressed as mean ± standard deviation *P<0.05, ** P < 0.01, *** P < 0.001, **** P<0.0001 compared with control- HC-402-05a cell line. B Results are expressed as mean ± standard deviation *P<0.05, ** P < 0.01, *** P < 0.001, **** P<0.0001 compared with control- hiPSCs cell line. (DOCX) [file pone.0205691.s003.docx]

A.

| **HC-402-05a** | **1 Gy** | | | |
| --- | --- | --- | --- | --- |
|  | 1h | 5h | 9h | 24h |
| **vs. hiPSCs** | ns | ns | ns | ** |
| **vs. hiPSC-DCHs** | ns | * | * | * |
| **P value summary** | ns | ns | * | * |
| **HC-402-05a** | **2 Gy** | | | |
|  | 1h | 5h | 9h | 24h |
| **vs. hiPSCs** | ns | ns | ns | ** |
| **vs. hiPSC-DCHs** | ns | ** | ** | ns |
| **P value summary** | ns | ** | ** | ** |
| **HC-402-05a** | **5 Gy** | | | |
|  | 1h | 5h | 9h | 24h |
| **vs. hiPSCs** | ns | * | ** | * |
| **vs. hiPSC-DCHs** | ns | ns | ** | ns |
| **P value summary** | ns | * | ** | ** |

B.

| **hiPSCs** | **1 Gy** | | | |
| --- | --- | --- | --- | --- |
|  | 1h | 5h | 9h | 24h |
| **vs. HC-402-05a** | ns | ns | ns | ** |
| **vs. hiPSC-DCHs** | ns | ns | ns | ns |
| **P value summary** | ns | ns | * | * |
| **hiPSCs** | **2 Gy** | | | |
|  | 1h | 5h | 9h | 24h |
| **vs. HC-402-05a** | ns | ns | ns | ** |
| **vs. hiPSC-DCHs** | ns | * | * | ns |
| **P value summary** | ns | ** | ** | ** |
| **hiPSCs** | **5 Gy** | | | |
|  | 1h | 5h | 9h | 24h |
| **vs. HC-402-05a** | ns | * | ** | * |
| **vs. hiPSC-DCHs** | ns | ns | ns | ** |
| **P value summary** | ns | * | ** | ** |

.
