# Supplementary material for: Chondrocytes differentiated from human induced pluripotent stem cells: Response to ionizing radiation
Source: PLoS One. 2018 Oct 23;13(10):e0205691. doi: 10.1371/journal.pone.0205691 (PMC6198947; doi:10.1371/journal.pone.0205691)
Supplement: S6 Table — Abbreviations: BRCA2 indicates breast cancer 2; RAD51, RAD51 recombinase; PRKDC, DNA-dependent protein kinase catalytic subunit; XRCC4, X-ray repair complementing defective repair in Chinese hamster cells 4; and PRKDC, DNA-dependent protein kinase catalytic subunit. (DOCX) [file pone.0205691.s007.docx]

| Gene | Primer sequence | Probe |
| --- | --- | --- |
| *BRCA2* | Forward: cctgatgcctgtacacctctt | 45 |
|  | Reverse: gcaggccgagtactgttagc |  |
| *RAD51* | Forward: atcactaatcaggtggtagctcaa | 58 |
|  | Reverse: cccctcttcctttcctcaga |  |
| *PRKDC* | Forward: agaggctgggagcatcact | 31 |
|  | Reverse: caccaaggcttcaaacacaa |  |
| *XRCC4* | Forward: tggtgaactgagaaaagcattg | 68 |
|  | Reverse: tgaaggaaccaagtctgaatga |  |
